# Describing the content of primary care: limitations of Canadian billing data

**DOI:** 10.1186/1471-2296-13-7

**Published:** 2012-02-15

**Authors:** Alan Katz, Gayle Halas, Michael Dillon, Jordan Sloshower

**Affiliations:** 1 Department of Family Medicine, Faculty of Medicine, University of Manitoba, Winnipeg, Manitoba, Canada; 2 Manitoba Centre for Health Policy, Winnipeg, Manitoba, Canada; 3 Department of Community Health Sciences, Faculty of Medicine, University of Manitoba, Winnipeg, Manitoba, Canada; 4 Klinic Community Health Centre, Winnipeg, Manitoba, Canada

**Keywords:** Primary care, ICD-9 code, Office visit, Topic, Action

## Abstract

**Background:**

Primary health care systems are designed to provide comprehensive patient care. However, the ICD 9 coding system used for billing purposes in Canada neither characterizes nor captures the scope of clinical practice or complexity of physician-patient interactions. This study aims to describe the content of primary care clinical encounters and examine the limitations of using administrative data to capture the content of these visits. Although a number of U.S studies have described the content of primary care encounters, this is the first Canadian study to do so.

**Methods:**

Study-specific data collection forms were completed by 16 primary care physicians in community health and family practice clinics in Winnipeg, Manitoba, Canada. The data collection forms were completed immediately following the patient encounter and included patient and visit characteristics, such as primary reason for visit, topics discussed, actions taken, degree of complexity as well as diagnosis and ICD-9 codes.

**Results:**

Data was collected for 760 patient encounters. The diagnostic codes often did not reflect the dominant topic of the visit or the topic requiring the most amount of time. Physicians often address multiple problems and provide numerous services thus increasing the complexity of care.

**Conclusion:**

This is one of the first Canadian studies to critically analyze the content of primary care clinical encounters. The data allowed a greater understanding of primary care clinical encounters and attests to the deficiencies of singular ICD-9 coding which fails to capture the comprehensiveness and complexity of the primary care encounter. As primary care reform initiatives in the U.S and Canada attempt to transform the way family physicians deliver care, it becomes increasingly important that other tools for structuring primary care data are considered in order to help physicians, researchers and policy makers understand the breadth and complexity of primary care.

## Background

Primary care practice provides comprehensive patient care including assessment, diagnosis and management of both acute and chronic problems. However, the ICD 9 coding system used in Canada does not capture the scope of disease or recognize the complexity of physician patient interactions. Administrative claims data in Canada use a single ICD-9 or ICD-10 code which does not elaborate on the severity of the disease, complexity of care or describe the extent of clinical management provided by the physician [1,2]. Administrative claims data are most useful for providing a picture of the population's health problems and the functioning of the health care system, such as estimates of disease prevalence and services provided to patients (e.g. Canadian Institute for Health Information [3]) rather than details of individual, patient level reports of care.

In the U.S, numerous studies have investigated the wide array of problems addressed and treatments provided during primary care encounters [4-8]. There have not been any Canadian studies to complement this data and therefore little is known about whether contextual differences such as funding mechanism or the predominance of family physicians in providing primary care alter the content of primary care encounters or the breadth of care provided by these physicians.

This study was initiated by a team of academic researchers and community-based family physicians with an interest in exploring the complexity of community-based care. In describing the content of primary care clinical encounters, this study's objectives were to identify the types of problems addressed by primary care physicians in a typical patient visit and to determine the kinds of actions taken and the various patient-related factors that influence visit complexity. These details are critically important in characterizing family practice, and in setting relevant educational, research, and policy priorities [7].

## Methods

### Setting

A convenience sample comprised of 16 primary care physicians collected data from five different primary care clinics in an urban center. Two of the study sites are community health clinics funded on salary, one is a university-based student clinic with a blended funding mechanism, one is a teaching clinic with salaried physicians and one is a private fee for service practice. The study was approved by the University of Manitoba Health Research Ethics Board.

### Data collection

This study utilized a specifically designed data collection form (Additional file 1: Appendix A) which was based on the National Ambulatory Medical Care Survey [9]. It was then pilot tested and revised through multiple iterations by the Manitoba Primary Care Research Network members. Once physicians were recruited to participate, they received written and verbal information which briefly explained the study purpose, procedures and directions for completing each section of the data collection form. They also determined a starting day and thereafter completed one form (immediately following the visit) for each consecutive patient encounter. Each full-time physician was asked to aim for a total of 100 forms or proportionately less if practicing part-time. The data collected includes general patient demographics; topics discussed and actions taken during the visit and an impression of the overall complexity of the visit. The physicians were also asked to identify which topics they considered to be the dominant one for each visit and which ones took the most time. One ICD-9 code was submitted for each patient encounter for billing or administrative purposes as per usual practice in Canada and this code was also documented by the physician on the data collection form.

### Data analysis

Univariate descriptive analyses were conducted to describe the content of the primary care encounter in terms of the number of topics discussed per visit, number of actions taken per visit, degree of complexity of patient visits and ICD-9 code submitted for each encounter. Logistic regression analysis was conducted to examine factors contributing to the complexity of the encounter. Analyses were conducted using the Statistical Package for Social Sciences 16.0.

## Results

A total of 760 data collection forms were completed. The mean number of data collection forms returned by each physician was 47 but ranged from 9 to 114. In 85.1% of cases, the physicians reported they were responsible for the ongoing care of the patient. Female patients made up 71.5 percent of the total sample, while 76 percent of all the patients had some post -secondary education. The average age of the patients was 41; 18.6% were older than 65 years of age.

### Topics

**Figure 1:**
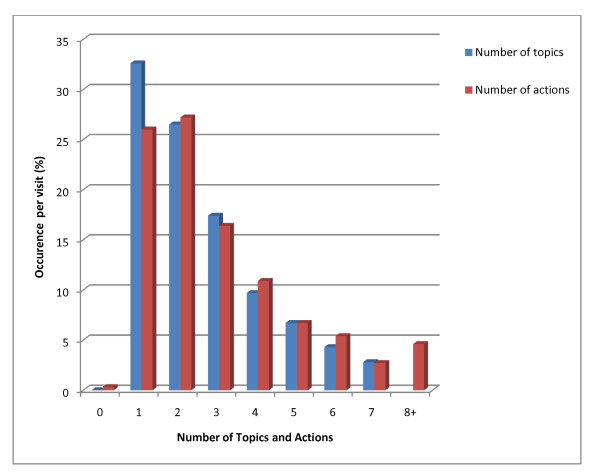
**Number of topics and actions per visit**.

The primary reasons for the visit are listed in Table 1. An average of 2.6 topics were addressed per patient visit, increasing to 3.3 topics among patients over 65 years old with 67.4% of patients having had more than one topic addressed (Figure 1). The majority of clinical encounters were initiated by patients, (rather than being requested by the physician) however this did not statistically impact the number of topics discussed at the visit. Patients initiated the discussion of the perceived "dominant topics" of visits more often than physicians. Patient-initiated visits were more likely to be periodic health exams or acute/episodic visits whereas physicians were more likely to initiate follow up care or prenatal care visits (*p *< .001).

**Table 1:** Primary reason for visit

	Frequency	Percent
Acute/episodic visit	269	35.4

Scheduled follow up	263	34.6

PHE	86	11.3

Chronic disease management	64	8.4

Prenatal care	20	2.6

Other	20	2.6

Counseling visit	6	0.8

Well child/immunization	5	0.7

Missing	27	3.4

Total	760	99.8

### ICD 9 coding

According to the ICD-9 codes recorded, periodic health evaluations, depression, hypertension, anxiety, diabetes mellitus, and fertility control were the most commonly discussed topics. This is consistent with administrative claims data with nine of the ten most frequently reported diagnoses being common to the two data sources (Katz A: Primary Care Diagnoses in Administrative Data, unpublished).

In this study, the ICD-9 code submitted for administrative purposes was found to match what physicians considered being the "dominant topic of the visit" for only 71% of patient visits. The ICD-9 code only matched 61% of topics that were considered to be the "topic requiring the most time" and was consistent with both the "dominant topic of the visit" and the "topic requiring the most time" for 58% of encounters. Table 2 presents the relationship between the dominant topic, topic requiring the most time or both dominant and time-consuming.

**Table 2:** ICD 9 Clusters recorded for those listed 10 or more times

	Frequency listed as ICD-9 Code	IC D-9 code also listed as dominant topic of the visit (%)	ICD 9 code also listed as topic requiring most time (%)	Number of topics discussed during the visit with this code (Mean)	Number of topics discussed during the visit (Range)
General Medical Examination	97	46.4	40.2	3	1-6

Nonpsychotic Depression	48	75.0	64.6	3	1-6

Anxiety/Neuroses	41	43.9	41.5	2	1-6

Hypertension	43	58.1	44.2	2	1-4

Diabetes Mellitus	32	75.0	50.0	4	1-6

Contraception	31	80.6	77.4	2	1-2

Prenatal and Postnatal Care (includes Complicated Pregnancy and Abortion)	21	71.4	71.4	2	1-6

Low Back Pain Diseases and Syndromes (exc acute strains)	21	90.5	76.2	2	1-5

Abdominal Pain (exc pelvic pain)	20	90.0	90.0	2	1-4

Acute Upper Respiratory Infection	10	90.0	70.0	2	1-2

Urinary Tract Infection (exc urethritis)	10	100.0	90.0	2	1-2

### Complexity of the encounter

Physicians considered 10% of patient visits to be very complex and 48% moderately complex. Logistic regression was conducted to determine the factors associated with visit complexity as determined by the physician. The independent variables included the clinic site, visit-related factors (total number of topics discussed per visit; total number of actions taken and patient-related factors (age, gender, presence of social or cultural issues). The total number of topics discussed significantly increased the likelihood of a visit being evaluated as complex (Odds Ratio = 1.36, CI 1.11 ~ 1.68). Presence of social or cultural issues did not significantly increase the likelihood of a visit being evaluated as complex (OR = 1.63, CI 0.92 ~ 2.89) nor was patient gender associated with perceived complexity.

## Discussion

This study aimed to describe the content of a primary care clinical encounter and describe the breadth and complexity of primary care as well as the inadequacy of a single ICD-9 code required by and used in Canadian billing claims. The findings support previous research indicating that physicians commonly address multiple problems and provide numerous services during family practice outpatient visits. A mean of 2.56 topics were discussed per visit resulting in an average of over three actions. This finding corresponds with reports from previous studies in the US [4,6]. as well as other countries [10]. Beasley et al [4] reported an average of 3.05 problems among the general population with an increase to 3.88 problems among patients over 65 years of age. Several additional studies using direct observation reported an average of 1.8-2.7 problems among their general patient population but as many as 58% of visits underreported the number of problems encountered [6].

The number of topics discussed per visit is associated with physicians reporting a clinical encounter to be complex. Considering that 90% of patients visiting their family physician have more than one chronic condition, and as many as 50% have five or more [11], primary care visits are more complex than the current coding practice indicates.

Most chronic conditions are managed within family practice with a small percentage of actions resulting in a referral to a specialist. Comorbidity is frequently addressed in the primary care encounter and primary care providers are the case managers for most patients with comorbidities as specialist care is sought for uncommon conditions [12]. Primary care providers are aware of the growing complexity of care expected outside of specialist care. The likelihood of this leading to physician burnout has been suggested [13].

Our data collection form gave the physicians an opportunity to list up to seven topics addressed during the visit, however only a single ICD code is submitted in most Canadian reimbursement systems and should reflect the primary problem addressed. The ICD-9 code did not consistently correspond to the problems considered dominant during the patient visit. They also did not consistently correspond to the problems that take the most time to address in a visit. In addition, episodic/acute visits were the most prevalent type of visits yet ICD-9 codes for general medical examinations or chronic disease management were the most frequently used diagnostic codes. This lack of congruence between codes and topics appears to be strongly related to whether visits were more complex visits (with multiple problems covered) rather than less complex or more symptom focused (Table 2). However, the use of a single ICD-9 code per interaction may potentially overstate the prevalence of chronic disease management. While research using administrative databases often use algorithms which incorporate other data sources, such as hospital discharge data or prescription drug use to manage the shortcomings of ICD-9 coding, [14] these do not account for the complexity of any particular visit nor do they identify the most important clinical activities. Thus, the use of a coding system that is not sensitive to the breadth and complexity of primary care encounters (such as ICD-9 codes) contributes to a distorted understanding of primary care encounters.

ICD coding originated for the purpose of categorizing and coding morbidity for public health purposes and until the mid 1970's was the most common means of classifying morbidity data within primary care. However, with its disease-based structure, many of the vague and/or ill defined symptoms and non-disease conditions that present in primary care are difficult to code with ICD codes. The disadvantages in using ICD spurred the World Organization of Family Doctors (WONCA) to design a classification specific to primary care. The International Classification of Primary Care (ICPC) provides a more comprehensive and patient centred structure while also ordering the data according to episodes of care (or first to last presentation of an illness or event) [15]. Structuring the data by utilizing episodes of care may allow greater recognition of the complexity of primary care encounters as well as recognizing the relationship of episodes across encounters [16].

The comprehensive care that is provided by family physicians is of particular relevance as we move toward care that is oriented toward the patient's overall health rather than disease-centered care. A broader view of patient care must be considered if primary care is to be properly understood. Effective multimorbidity case management requires an approach that cannot be neatly categorized into a discrete ICD diagnostic code. A more holistic approach to caring for patients with multiple morbidities requires moving beyond codes or labels and considering varying levels of severity and emotional distress that accompany their conditions [17]. Furthermore, effective quality care considers the patients' concerns and requires time for careful listening, planning and negotiating - valued aspects of holistic care and greater patient satisfaction [18]. The patient-centered medical home concept prioritizes accessible, comprehensive coordinated medical care [19]. To advance this concept, there is a need for greater understanding of complexity measures [13] and related factors as well as greater specificity in how primary care is characterized within coding schemes. The limitations of ICD coding in describing the content of the primary care visit have become evident within the encounter data collected in this study. The shortcomings of ICD are further exacerbated by the current reporting of a single ICD code in Canada. Clearly, it is time for a coding system that will more accurately document the provision of patient care, disease-related complications and resource utilization in primary care [2]. Funding and system reform priorities set on the basis of administrative data alone cannot describe the complex management of patient problems within a context of competing demands and will likely miss significant primary care practice considerations.

### Limitations

While a multi-method approach, such as one that combines direct observation, medical record review, patient survey, and/or billing data would likely provide a more comprehensive, unbiased view of primary care clinical encounters, our results are consistent with other U.S studies which have used direct observation [6,7]. The short duration of the practice log does not provide longitudinal data which may address questions that cannot be answered here (such as seasonal variation for topics), and would enhance validity and generalizability of findings. Physician demographics and contextual patient data were not collected. This was a convenience sample of self-selected physicians which may have lead to participant bias. As a group of urban practitioners, the findings may not represent physicians in general. However, we might expect rural physicians to have an even broader scope of practice particularly where they are more distant from other specialists and/or allied health services.

## Conclusion

This is one of the first Canadian studies to critically analyze the content of primary care clinical encounters. The data allowed a greater understanding of primary care clinical encounters and attests to the deficiencies of singular ICD-9 or billing coding. Therefore, as primary care reform initiatives in the U.S and Canada attempt to transform the way family physicians deliver care, it becomes increasingly important that physicians, researchers and policy makers develop a thorough understanding of the breadth and complexity of primary care encounters.

## Competing interests

The authors declare that they have no competing interests.

## Authors' contributions

AK and MD were involved in the conception and design of the study. GH and JS were involved in data analysis and manuscript drafting with significant contributions from both AK and MD. All authors reviewed drafts, contributed to revisions and approved the final manuscript.

## Pre-publication history

The pre-publication history for this paper can be accessed here:

http://www.biomedcentral.com/1471-2296/13/7/prepub

## Supplementary Material

Additional file 1 **Appendix A Data collection form**. Click here for file
