# Supplementary material for: Describing the content of primary care: limitations of Canadian billing data
Source: BMC Fam Pract. 2012 Feb 15;13:7. doi: 10.1186/1471-2296-13-7 (PMC3305652; doi:10.1186/1471-2296-13-7)
Supplement: Additional file 1 — Appendix A Data collection form. [file 1471-2296-13-7-S1.PDF]

Physician Code_________ *Date of Visit:___________________* *Study Number:________________*

Manitoba Primary Health Care Research Network - Data Collection Form
"The Content of a Primary Care Clinical Encounter"

Do you take responsibility for care of this patient? Yes___ No___

Patient Demographics:

Age:_________ Gender:_________ Education Level**:** ___ >High School ___ <High School ___Don't Know

Relevant Social or Cultural Issues:____

Primary Reason for Visit:

___Scheduled Follow Up
___Chronic Disease Management
___Well Child/Immunization
___PHE
___Counselling Visit
___Acute/Episodic visit
___Prenatal Care
___Other: ______________________________________
____

___Patient Initiated Visit ___Physician Initiated Visit

Excluding this visit, how many times did you see this person in the last year?_____________

**Topic Discussed**	**Dominant Topic(s) of the Visit (√)** (please check only one or two)	**Topic requiring the most time? (√)**	**Initiated by: (check one)**		**Action(s) Taken** **R** – Referral (to what services?) **M** – Medication **F** – Follow up **C** – Counselling **PE** – Patient Education **O** – Other (please explain) **RR** – Reassurance/resolved
			Physician	Patient	

What ICD 9 code (or diagnosis) was submitted to Manitoba Health for billing purposes? __________

Which tariff(s) did you bill for this visit? __8529 Regional intermediate visit > 10 min with exam
__8509 Regional visit < 10 min (no exam)
__8540 Complete history & physical
__8400 Comprehensive prenatal assessment
__8401 Prenatal visit
__Other: ________________________________
____
__8498 Complete history & physical with gyne exam and pap
__8499 Complete history & physical with gyne exam without pap
__8470 Regional intermediate visit with gyne exam and pap
__8471 Regional intermediate visit with gyne exam without pap

What was the degree of complexity of this visit?
___ Not Complex ___ Moderately Complex ___ Very Complex
